# Incorporating Evolutionary Theory Into Forensic Anthropology Methods and Practice: A Proof‐of‐Concept Study Using Skeletal Sex Estimation

**DOI:** 10.1002/ajpa.70137

**Published:** 2025-10-07

**Authors:** An‐Di Yim, Michala K. Stock, Allysha P. Winburn

**Affiliations:** ^1^ Forensic Science Program George Mason University Manassas Virginia United States; ^2^ Department of Anthropology University of Illinois Urbana‐Champaign Urbana Illinois United States; ^3^ Department of Sociology and Anthropology Metropolitan State University of Denver Denver Colorado United States; ^4^ Department of Anthropology University of West Florida Pensacola Florida United States

**Keywords:** forensic anthropology, morphological integration, sex estimation

## Abstract

**Objectives:**

This study provides a proof‐of‐concept for incorporating evolutionary theory into forensic anthropology practice. Specifically, we test whether innominate measurements used in the DSP 2 sex‐estimation method reflect known patterns of morphological integration and whether variable redundancy can be reduced without compromising classification accuracy.

**Materials and Methods:**

Innominate measurements were obtained from published datasets totaling 3045 individuals. Principal component analysis (PCA) was used to identify clusters of measurements. Relative standard deviation of eigenvalues was used to assess the degree of morphological integration. Posterior probabilities of sex classification were computed using one variable per cluster (cluster‐based approach) and compared to a randomly selected four‐variable approach, consistent with the minimum recommended by the original study. Simulations were used to generate posterior distributions of accuracy and the percentage of samples reaching a decision threshold.

**Results:**

Three distinct clusters of innominate measurements were identified, broadly corresponding to known modules of the innominate. The degree of morphological integration was higher within clusters than in the full measurement set or nonintegrated matrices. The cluster‐based classification approach showed comparable accuracy (mean = 96.38%) to the randomized approach (mean = 95.64%) despite using only three variables. While fewer individuals were assigned a sex under the cluster‐based method, the results demonstrated higher consistency.

**Discussion:**

Results suggest that accounting for morphological integration can streamline sex estimation by reducing variable redundancy without compromising accuracy. This study demonstrates how evolutionary principles can improve the theoretical foundation of forensic anthropology methods and offers a framework for future method development grounded in evolutionary theory.

## Graphical abstract

This study shows measurements in the DSP2 sex‐estimation method cluster into integrated modules. Selecting one measurement per module preserves accuracy while reducing redundancy, demonstrating the value of incorporating evolutionary theory into forensic anthropology. Images derived from BodyParts3D. The Database Center for Life Science, licensed under CC BY‐SA 2.1 Japan.

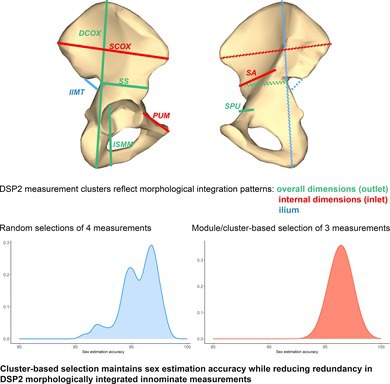

## 1 Introduction

Skeletal sex estimation is typically the first step in constructing a biological profile from skeletal remains. While most practitioners employ a variety of nonmetric and metric methods involving different skeletal regions (Klales 2020; Klales and Lesciotto 2025), the innominates remain the most reliable indicator of sex, followed by postcranial metrics and the skull (Klales et al. 2012; Spradley and Jantz 2011). Currently, nonmetric methods using innominate traits (i.e., Klales 2018; Klales et al. 2012) and metric methods incorporating multiple skeletal regions (i.e., Jantz and Ousley 2005) are the most preferred and commonly used in casework (Klales 2020; Klales and Lesciotto 2025).

*Diagnose sexuelle probabiliste* (DSP), a metric method introduced in 2005, estimates skeletal sex using 10 measurements of the innominate and a linear discriminant function based on a reference sample of more than 2000 innominates (Murail et al. 2005). The method was later developed into a freely available software package, DSP 2, and validated using a sample of ~600 innominates (Brůžek et al. 2017).

Most metric approaches in forensic anthropology treat each measurement as an independent unit. However, many innominate measurements are not independent due to shared developmental processes and genetic architecture. Traits tend to covary as the result of *morphological integration* (the tendency for morphological complexes to evolve as a cohesive unit), with subsets of traits that show more correlation with one another (*modularity*) (Hallgrímsson et al. 2009). Previous evolutionary and morphometric studies have shown that, while the human hip exhibited less integration and developmental constraint compared to other Great Apes, certain innominate measurements, such as those associated with the dimensions of the ischium, remain highly integrated (i.e., they are more correlated with one another than with other traits) (Grabowski et al. 2011). Traits such as innominate height, iliac breadth, and pubic length have also been shown to covary in response to climatic selection (Betti et al. 2014). Mallard et al. (2017) further demonstrated that the innominate comprises multiple modules, with more trait integration (i.e., a higher degree of correlation) within each module.

In justifying the selection of the 10 innominate measurements, the developers of DSP and DSP 2 acknowledged the non‐independence of skeletal traits, grouping these variables into two broader functional units: the sacroiliac region and ischiopubic region (Santos et al. 2020). However, this model is likely an oversimplification of the innominate morphological complex and does not take into account integration within and across these regions.

The validity of DSP 2 has been supported in a contemporary US sample, and its use for forensic casework has been recommended (Lesciotto and Klales 2025). Still, an assessment of correlations among these measurements used in DSP 2, especially those likely arising from shared developmental and evolutionary constraints, may help identify redundancy. Testing the accuracy of sex estimation when correlated (redundant) variables are removed from the estimation model could improve efficiency and inform future model design.

Previously, the authors of the current study called for the incorporation of evolutionary theory into our methods and practice (Winburn et al. 2022). This study responds to this call by offering a proof‐of‐concept framework that integrates evolutionary theory into forensic anthropology. Specifically, this study aims to test whether the patterns of correlation among innominate measurements used in the DSP 2 sex estimation model reflect known patterns of morphological integration in humans. We then assess the redundancy of these measurements, exploring whether the inclusion of highly correlated traits contributes to duplicated information in the context of sex estimation.

## 2 Materials and Methods

**FIGURE 1:**
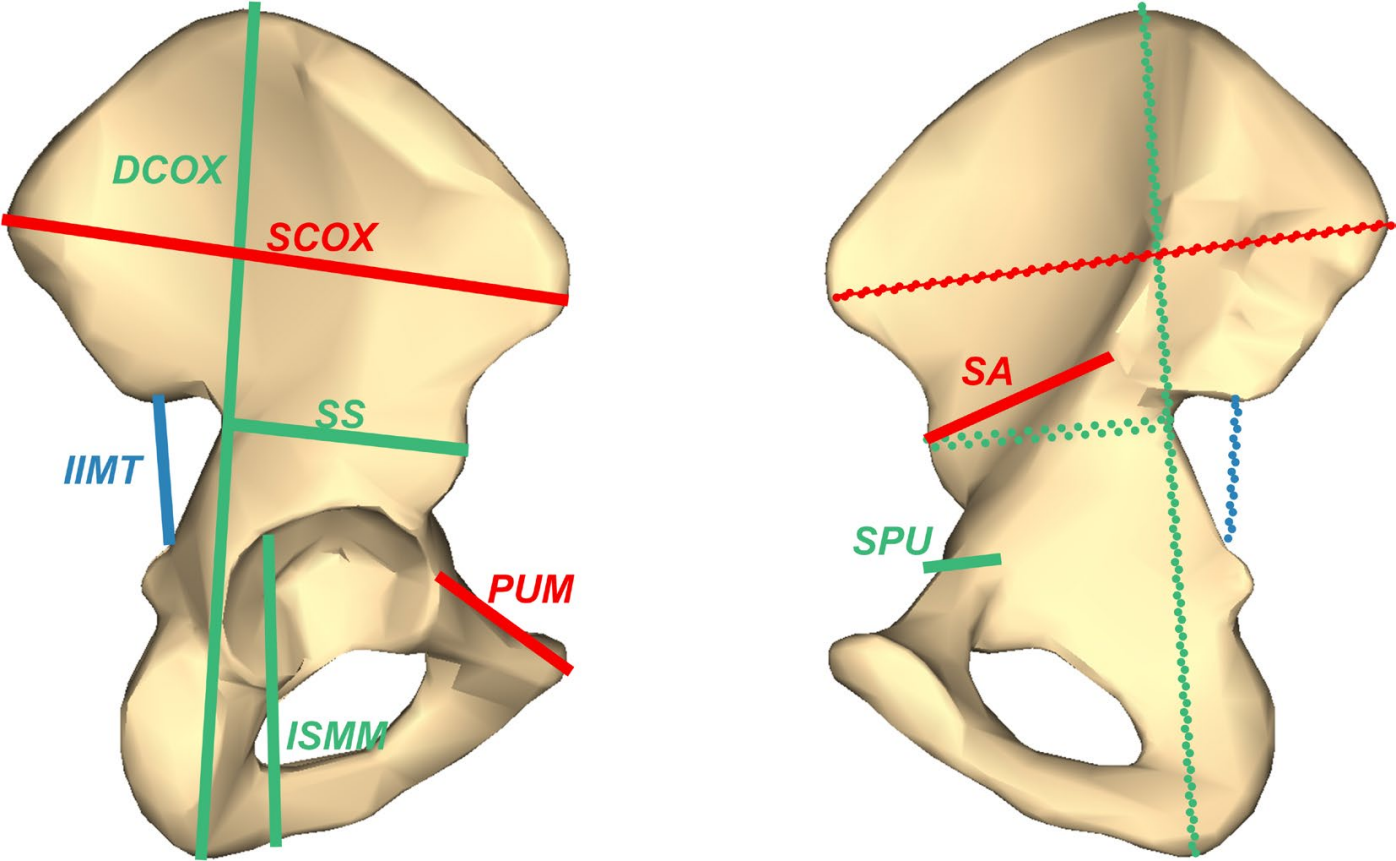
Depictions of the DSP 2 innominate measurements used in this study, approximated by the first author (for precise anatomical landmarks used in data collection, please see Brůžek et al. (2017) and Lesciotto and Klales (2025)). Solid lines indicate the observed measurements; dashed lines indicate the corresponding measurements on the opposite side of the element. Colors correspond to the trait clusters identified in this study: green = pelvic inlet module, red = pelvic outlet module, blue = ilium module. Innominate images derived from BodyParts3D. The Database Center for Life Science, licensed under CC BY‐SA 2.1 Japan.

The measurements used in DSP 2 include: PUM (acetabulo‐symphyseal pubic length), SPU (cotylo‐pubic width), DCOX (innominate height), IIMT (greater sciatic notch height), ISMM (post‐acetabular ischium length), SCOX (iliac breadth), SS (spino‐sciatic length), SA (spino‐auricular length), SIS (cotylo‐sciatic breadth), and VEAC (vertical acetabular diameter). Their definitions were described in detail elsewhere (Brůžek et al. 2017; Lesciotto and Klales 2025; Murail et al. 2005; Santos et al. 2020). SIS and VEAC were considered “rescue” measurements recommended only for incomplete innominates (Brůžek et al. 2017). Therefore, these two variables were excluded from our analysis. The approximate locations of the eight measurements are depicted in Figure 1; however, it should be noted that these are author‐drawn guides and not intended to represent the precise anatomical landmarks used in data collection (see Brůžek et al. 2017 and Lesciotto and Klales 2025 for detailed definitions). According to Santos et al. (2020), DCOX and SCOX represent overall innominate dimensions, while the remaining six measurements represent two functional modules: PUM, SPU, and ISMM are grouped together as the ischio‐pubic module, and IIMT, SS, and SA comprise the sacro‐iliac module.

Osteometric data were extracted from published studies (Brůžek et al. 2017; Kuchař et al. 2024; Machado et al. 2018), resulting in a total of 3045 sets of innominate measurements. For analyses requiring complete data (principal component analysis (PCA) and morphological integration analysis), we used complete observations only. A total of 1855 sets (941 males and 944 females) from Brůžek et al. (2017) were used as the reference/training dataset. The dataset from Kuchař et al. (2024) included 272 sets of complete innominate measurements but lacked associated demographic information. Therefore, this dataset was used as the validation set for morphological integration analysis. For sex classification validation, we used a combined 727 sets of innominate measurements (367 males and 360 females) from Brůžek et al. (2017) and Machado et al. (2018) with varying patterns of missingness: PUM (313 missing), SPU (200), DCOX (149), IIMT (15), ISMM (128), SCOX (220), SS (12), and SA (12). No data imputation was performed.

In analyzing integration as a result of genetic and developmental underpinnings, the use of the genetic variance–covariance matrix (**G**) is required. However, estimating **G** can be challenging as it requires phenotyping and genotyping a large number of individuals or access to known pedigree information. In most cases, a common and practical assumption is that the phenotypic variance–covariance matrix (**P**) provides a good proxy for **G**, particularly for morphological traits that tend to exhibit moderate‐to‐high heritabilities and are often influenced by pleiotropic effects (Cheverud 1988; Rolian 2014). This assumption is appropriate for innominate metric data, as the average heritability across all innominate measurements was estimated to be ~0.4 (Xu et al. 2025). The only previously published study on genetic correlations among human innominate traits (Sharma 2002) also reported high levels of heritability. However, the study design (i.e., twin study) was demonstrated to have trouble separating common environmental components of phenotypic variance from genetic components (Kruuk 2004; Kruuk and Hadfield 2007) and should be interpreted with caution. Nonetheless, in the present context, **P** could be considered a reasonable substitute for **G**.

PCA was used to identify clusters of traits, and the relative standard deviation of eigenvalues (Pavlicev et al. 2009) was used to quantify the degree of morphological integration within and between these clusters of traits. Rather than using the DSP 2 software, we implemented the DSP 2 classification scheme using custom code in the R Programming Language (R Core Team 2025) following the methods outlined in Brůžek et al. (2017). For each sample of the validation datasets, one measurement was randomly selected from each identified cluster, and posterior probabilities of sex classification were calculated. The cut‐off for assigning biological sex was set at 0.85, in line with more recent studies (Avent et al. 2022; Jerković et al. 2020), instead of the 0.95 threshold originally recommended by Brůžek et al. (2017). This process was repeated 10,000 times to provide a posterior distribution of accuracy rates for the cluster‐based approach. To compare the performance of the cluster‐based approach, we simulated the random drawing of four variables (without regard to cluster structure) for each sample in the validation dataset. Posterior probabilities for sex classification were then calculated using the same threshold. This process was repeated 10,000 times to provide a null distribution of accuracy rates. For each simulation iteration (for both cluster‐based and randomized approaches), individuals were only included if they had complete values for the selected set of variables. Individuals missing one or more of the selected variables were excluded for that iteration, but could be included in subsequent iterations if the required variables were present. All statistical analyses were performed using R version 4.4.3 (R Core Team 2025).

## 3 Results

**FIGURE 2:**
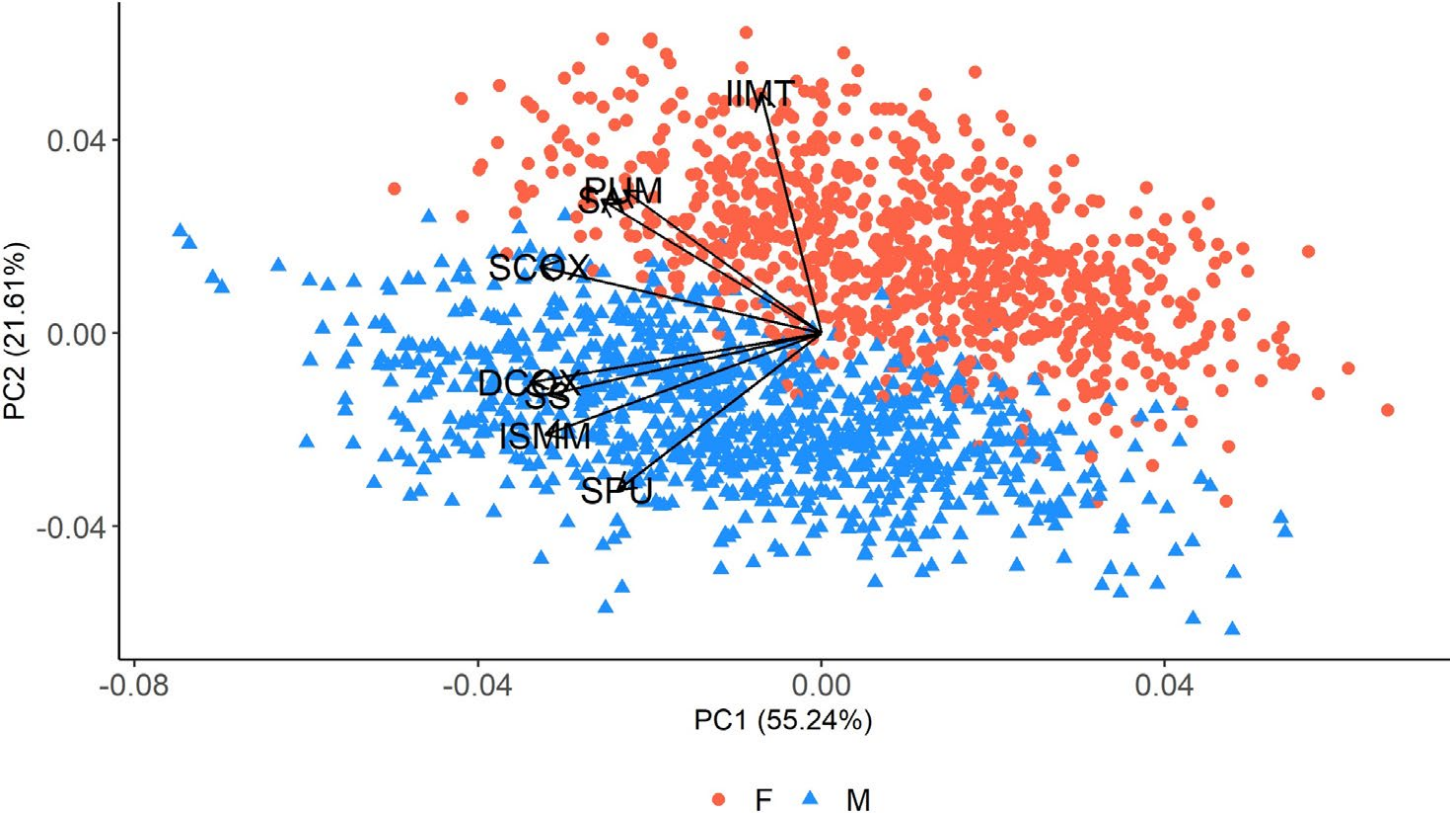
Principal component analysis (PCA) biplot of the reference dataset showing the direction of eigenvectors for DSP 2 osteometric variables and their clustering patterns. IIMT forms a separate loading vector, suggesting weak correlation with other variables. PUM, SA, and SCOX form a distinct cluster, indicating shared variation. The remaining variables (DCOX, SS, ISMM, and SPU) form a dense cluster, suggesting a high degree of correlation. Points are colored by biological sex (red = female, blue = male) and distinguished by shape for visualization.

**FIGURE 3:**
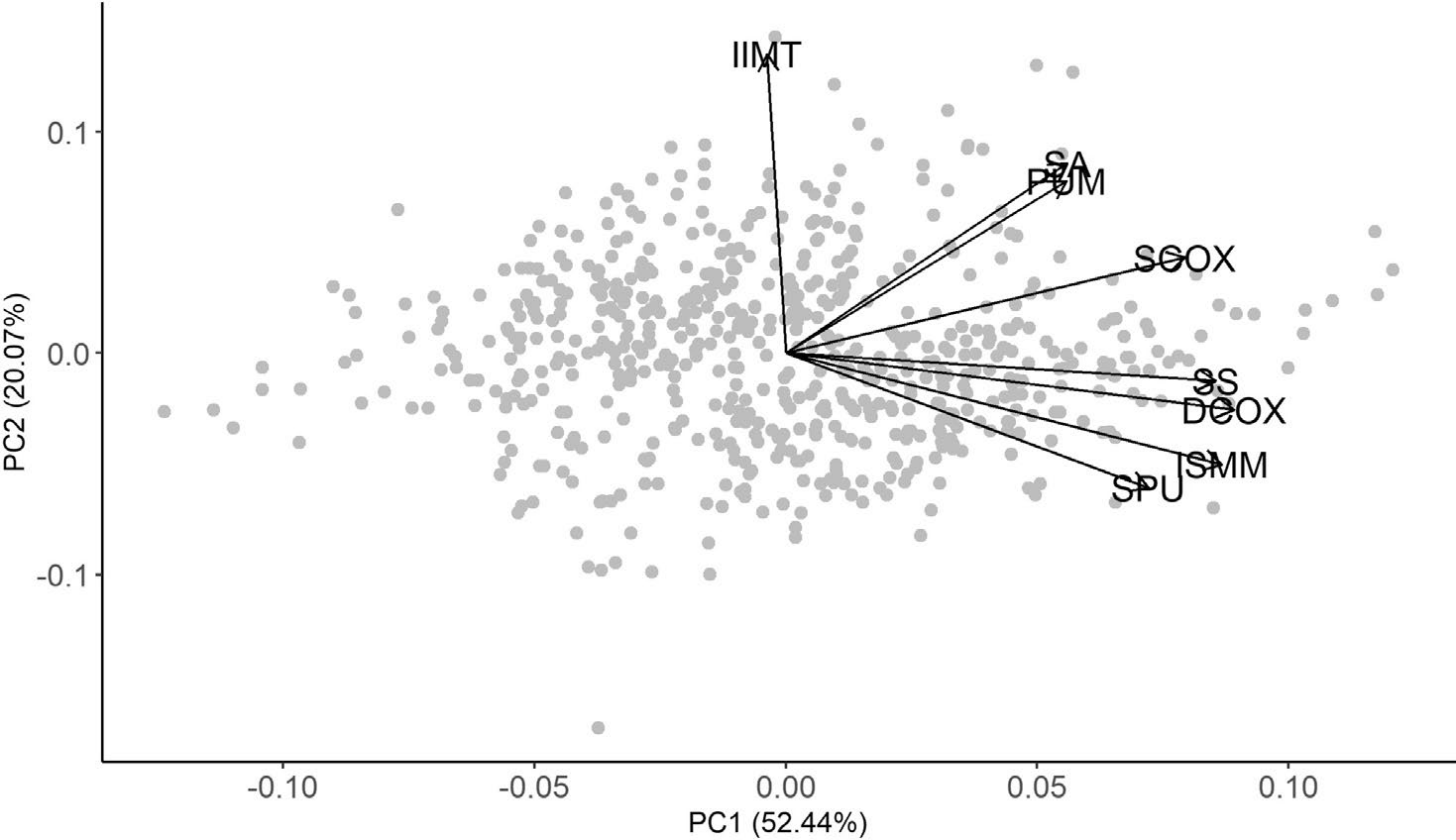
Principal component analysis (PCA) biplot of the Kuchař et al. (2024) datasets. The structure mirrors that of the reference dataset, providing support for the consistent trait covariation structure observed among DSP 2 pelvic measurements.

PCA reveals distinct clusters among the DSP 2 innominate measurements, likely reflecting underlying patterns of morphological integration. The PCA biplot of the reference dataset (Figure 2) shows that IIMT forms an isolated loading vector, suggesting low correlation with other variables. PUM, SA, and SCOX form a second cluster, indicating shared variation likely associated with the overall breadth of the pelvis. The remaining variables (DCOX, SS, ISMM, and SPU) form another cluster, suggesting high internal correlation and likely developmental and/or functional integration. These clusters broadly align with known modules of the innominate (innominate shape, pelvic outlet, and pelvic inlet), providing a framework for assessing redundancy in sex estimation models. PCA of the validation dataset (Figure 3) reveals a highly consistent clustering pattern compared to the reference data, further supporting the trait covariation structure observed among DSP 2 measurements. The replication of this structure across datasets underscores the biological relevance of these modules and supports the interpretation of trait redundancy based on morphological integration. The degree of morphological integration among DSP 2 measurements is presented in Table 1. As expected, the clusters identified via PCA show greater morphological integration than the full set of DSP 2 measurements or a random correlation matrix of nonintegrated traits (null hypothesis), supporting their use as cohesive units.

**TABLE 1:** Magnitude of morphological integration among DSP 2 measurement as represented by the relative standard deviation of eigenvalues (${\mathrm{SD}}_{\mathrm{rel}}\left(\lambda \right)$). The observed relative standard deviation for each cluster is compared to the expected relative standard deviation of eigenvalues under a null hypothesis of no integration (random correlation matrix, $\sqrt{1/2M}$).

Innominate set	Sample size	# of traits	${\mathrm{SD}}_{\mathrm{rel}}\left(\lambda \right)$	$\sqrt{1/2M}$
All	1885	8	0.5225	0.0163
Pelvic outlet (PUM, SA, SCOX)	1967	3	0.6120	0.0159
Pelvic inlet (DCOX, SS, ISMM, SPU)	1989	4	0.7280	0.0159

**FIGURE 4:**
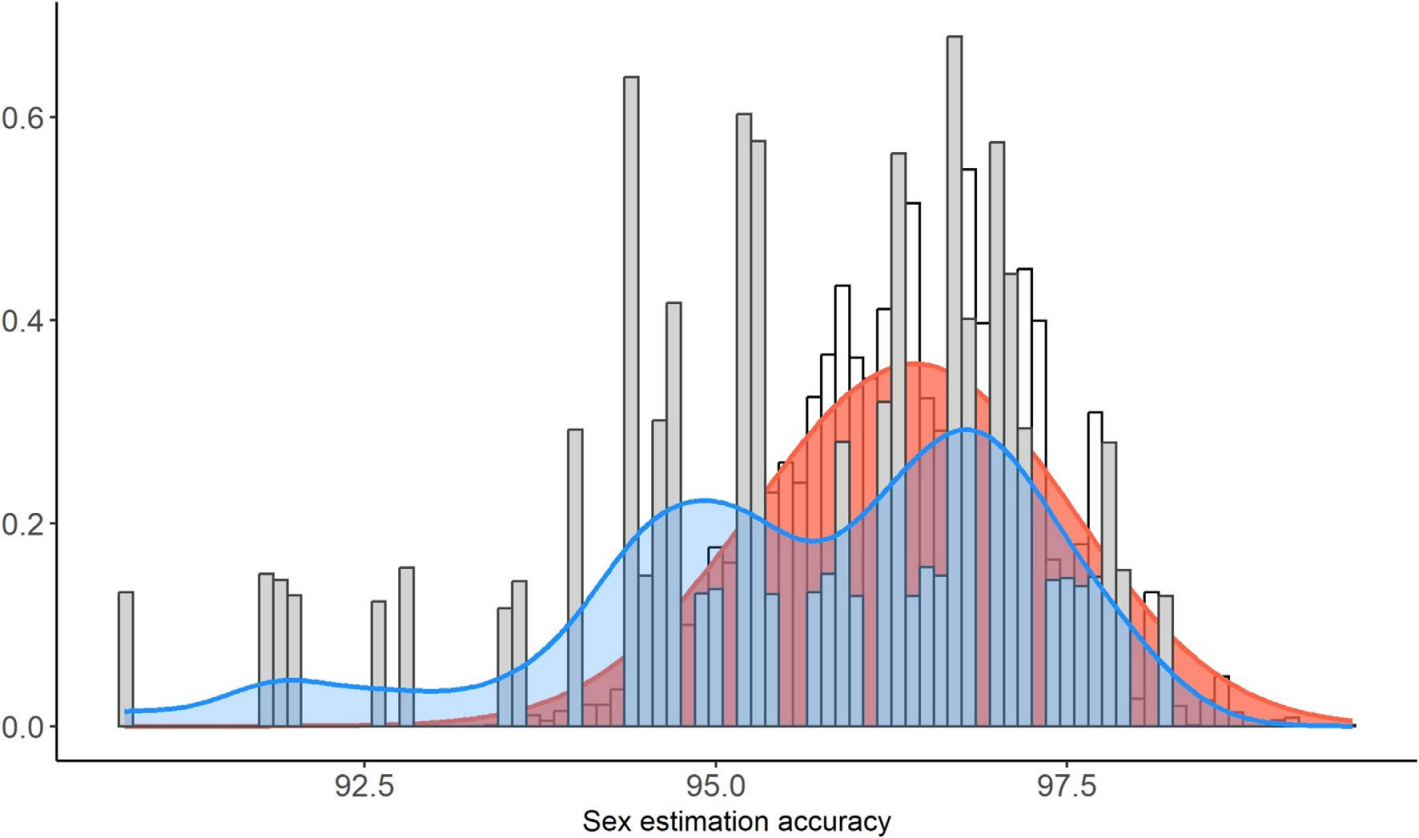
Distribution of sex estimation accuracy from 10,000 simulations comparing the cluster‐based approach (red) to the randomly selected four‐variable approach (blue). The cluster‐based method achieves higher accuracy (mean accuracy: 96.38% vs. 95.65%) with more consistency in performance. This supports the hypothesis that selecting one measurement per morphologically integrated cluster maintains classification performance while reducing redundancy in input variables.

Figure 4 shows the distribution of sex estimation accuracy from 10,000 simulations using a posterior probability threshold of 0.85, comparing the performances of the cluster‐based approach (one measurement randomly selected from each identified cluster) with that of the randomized four‐variable approach (a random drawing of four variables) for the validation data set. The cluster‐based approach achieved higher accuracy (mean accuracy: 96.38%) with greater consistency in performance compared to the randomized approach (mean accuracy: 95.64%). A two‐sample Kolmogorov–Smirnov test showed a significant difference between approaches (D^+^ = 0.3106, *p* < 2.2 × 10^−16^). This indicates that, across simulations, the cluster‐based method generally yielded higher accuracy values than the randomized approach.

**FIGURE 5:**
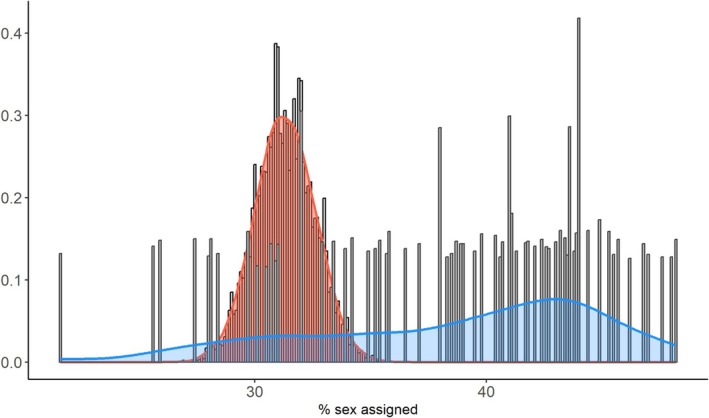
Distribution of the percentage of individuals reaching the posterior probability threshold of 0.85 across 10,000 simulations. The cluster‐based approach (red, mean = 31.31%) yields a narrower, more centralized distribution, indicating more consistent classification performance. In contrast, the random selection approach (blue, mean = 38.56%) shows greater variability, suggesting greater sensitivity to variable choice.

Because IIMT forms its own cluster, it was always included in the cluster‐based approach. Variable selection in both approaches was influenced by differences in missingness across measurements, but the large number of simulations should minimize this effect. Figure 5 shows the distribution of the percentage of innominates successfully assigned a sex across the same simulations. The cluster‐based approach resulted in a narrower, more centralized, and consistent distribution (mean: 31.31%), while the randomized approach showed greater variability in assignment rates (mean: 38.56%), suggesting greater sensitivity to variable choice. While the overall percentage of sex assigned innominates under the cluster‐based approach was lower, this is likely due to the use of only three variables rather than the four variables minimally recommended in the DSP 2 protocol and highlights a practical limitation of the current cluster‐based approach.

## 4 Discussion

This study served as a proof‐of‐concept for integrating evolutionary theory into forensic anthropology by evaluating whether a morphological integration‐informed approach can improve the efficiency of metric sex estimation and inform future model design. Specifically, we examined the patterns of phenotypic correlation among innominate measurements used in the DSP 2 sex estimation model, assessing their consistency with known patterns of morphological integration in humans, and assessed the accuracy of sex estimation when correlated (redundant) variables were removed. Echoing the results of previous studies (Brůžek et al. 2017; Santos et al. 2020), we found distinctive clusters among the DSP 2 innominate measurements and replicated these results using a validation sample. We also found greater degrees of morphological integration within clusters compared to the full set of DSP 2 measurements or a random correlation matrix of nonintegrated traits, supporting their interpretation as cohesive morphological units. Finally, the classification accuracy of the cluster‐based approach, randomly selecting one measurement from each identified cluster, was shown to achieve higher accuracy than that of using four randomly selected measurements, though with lower assignment rates. The results support the hypothesis that selecting one representative measurement per morphologically integrated cluster maintains classification performance while reducing redundancy in input variables.

The clusters identified here broadly align with previous findings (Betti et al. 2014; Mallard et al. 2017), with measurements grouped into modules related to the overall pelvic dimensions (PUM, SA, and SCOX, pelvic outlet) and internal pelvic dimensions (DCOX, SS, ISMM, SPU, pelvic inlet). Greater sciatic notch height (IIMT) formed an isolated grouping, likely because variation in this measurement affects the overall orientation and shape of the ilium and thus does not cluster with other modules. The degree of morphological integration was greater within these clusters than across the full set of DSP 2 measurements or within a random correlation matrix of nonintegrated traits. While the calculation of relative standard deviation of eigenvalues can be inflated by a reduced number of traits (Mallard et al. 2017; Pavlicev et al. 2009), this potential bias was likely offset by the large sample size used in this study (Haber 2011). Notably, the “best four” variables identified by Brůžek et al. (2017) in the original DSP 2 study also map closely onto the modules reported here: PUM within the pelvic outlet module, DCOX and SPU within the pelvic inlet module, and IIMT in the isolated ilium module. This consistency suggests that DSP2's most informative variables inherently reflect patterns of morphological integration in the innominate, providing an evolutionary explanation for their utility and offering a framework for evaluating the performance of reduced‐variable sex‐estimation approaches.

The classification accuracy using the cluster‐based approach was comparable to that of using four randomly selected variables without regard to morphological modules. This is broadly consistent with the accuracy rates (at least 96%) reported by the original publication (Brůžek et al. 2017), previous findings using different combinations of variables (a combined 95% accuracy using all 10 measurements, Quatrehomme et al. 2017), and aligns with the most recent validation study using a contemporary US sample (at least 95% accuracy using 10 measurements, Lesciotto and Klales 2025). Compared with a previous study of metric sex estimation accuracy using casework data (Thomas et al. 2016), the performance of DSP 2 is notably improved, even with just three measurements. While the percentage of individuals assigned a sex using the cluster‐based approach was lower (average in the low 30%) compared to the randomized four‐variable approach (mid‐20% to mid‐40%), this is likely due to the use of fewer input variables. The low classification rates even with lower posterior probability thresholds, compared to the original study's findings and the findings of Quatrehomme et al. (2017) (both ~94% classification rates) using the full set of DSP 2 measurements, represent the limitation of applying the cluster‐based approach to casework without further model or method improvements. These findings reported in this study, however, suggest that selecting traits from distinct morphological modules can enhance classification efficiency without sacrificing accuracy, and that adding one or more representative traits from different modules could further improve model performance. It is also important to note that DSP2 is not population specific. This highlights the broad relevance of our findings, as patterns of morphological integration in the innominate are expected to remain consistent across human populations.

While the DSP 2 innominate measurements have generally acceptable intra‐ and inter‐observer errors (Lesciotto and Klales 2025; Machado et al. 2018; Quatrehomme et al. 2017), most are not part of the standard measurements defined in the *Standards for Data Collection from Human Skeletal Remains* (Buikstra and Ubelaker 1994) or *Data Collection Procedures for Forensic Skeletal Material 2.0* (Langley et al. 2016), and only two of the measurements are used by the program Fordisc (Jantz and Ousley 2005). In their evaluation, Lesciotto and Klales (2025) reported that these measurements are “relatively easy to take” with minimal technical requirements and recommended their use in casework, especially in situations where alternative methods yield ambiguous results, since the classification algorithm of DSP 2 is robust and its accuracy rate is high. We recommend that future work aimed at improving the DSP 2 model focus on identifying innominate measurements that both reflect morphological modules that can be taken with ease and with consistent intra‐ and inter‐observer reliability.

The results of the cluster‐based approach presented in this study could be further improved by adjusting the posterior probability threshold, as suggested by Jerković et al. (2020). For example, when a threshold of 0.75 was used, ~60% of the validation samples were assigned a sex while the classification accuracy remained the same. However, setting a strict threshold implicitly treats sex as a fixed binary category. In contrast, the use of posterior probabilities directly reframes sex as a probabilistic trait derived from the morphometric space of multiple innominate measurements. When used in this regard, sex‐estimation methods can more effectively acknowledge the biological complexity and continuous variation in human skeletal morphology (Meloro et al. 2025). Given that practitioners routinely use multiple methods in casework (Klales 2020; Klales and Lesciotto 2025), a more flexible approach may be to simply report the posterior probabilities from different methods directly, rather than using a strict threshold. This allows practitioners to interpret skeletal morphology as a continuum, informed by both context and probability.

Using a cluster‐based approach to sex estimation may also prove useful in cases where minimizing the number of skeletal measurements is a priority. In contexts of forensic humanitarian work and bioarcheological analyses of ancestral remains, for example, descendant stakeholders may wish for the handling of remains to be minimized or completed in a timely manner. In such cases, subjecting skeletal individuals to fewer measurements would be ideal. This preliminary research indicates that such an approach can maintain accuracy with fewer variables, though at the cost of classifying fewer individuals, as long as metric traits are chosen with an eye to morphological integration.

In summary, this study demonstrates that incorporating concepts of morphological integration into forensic anthropological methods can enhance the efficiency and theoretical foundation of sex estimation methods and guide future method design. By recognizing and accounting for trait covariation, practitioners can streamline metric approaches without compromising accuracy. While our cluster‐based approach maintained high accuracy, the lower proportion of individuals classified represents a key limitation that currently restricts direct casework application. Forensic anthropologists should therefore continue to use DSP2 with as many available measurements as possible. However, when remains are fragmentary, prioritizing at least one measurement from each morphological module identified here may help maximize classification potential. As forensic anthropology continues to evolve, approaches grounded in evolutionary theory offer promising ways to refine and improve our methods and practices. Moreover, although we present this work as a response to our previous call to integrate evolutionary theory into forensic anthropology practice, its relevance extends far beyond forensic applications, offering a framework that is equally valuable to biological anthropologists studying human variation and evolution.

## Author Contributions

**An‐Di Yim:** conceptualization, funding acquisition, writing – original draft, writing – review and editing, formal analysis, investigation, data curation, visualization. **Michala K. Stock:** conceptualization, writing – review and editing, writing – original draft, formal analysis, funding acquisition. **Allysha P. Winburn:** writing – review and editing, conceptualization, writing – original draft, formal analysis, funding acquisition.

## Conflicts of Interest

The authors declare no conflicts of interest.

## Data Availability

All data used in support of this publication are publicly available through previously published sources cited in the manuscript. These datasets can be accessed in the supporting information of the following publications: https://doi.org/10.1002/ajpa.23282, https://doi.org/10.1016/j.forsciint.2018.06.043, and https://doi.org/10.1007/s00414‐024‐03301‐4. The R code used in this manuscript is available in the first author's GitHub repository: https://github.com/andicyim/DSP2_replication.

## References

[ajpa70137-bib-0001] Avent, P. R. , C. E. Hughes , and H. M. Garvin . 2022. “Applying Posterior Probability Informed Thresholds to Traditional Cranial Trait Sex Estimation Methods.” Journal of Forensic Sciences 67, no. 2: 440–449. 10.1111/1556-4029.14947. 34799862

[ajpa70137-bib-0002] Betti, L. , N. von Cramon‐Taubadel , A. Manica , and S. J. Lycett . 2014. “The Interaction of Neutral Evolutionary Processes With Climatically‐Driven Adaptive Changes in the 3D Shape of the Human Os Coxae.” Journal of Human Evolution 73: 64–74. 10.1016/j.jhevol.2014.02.021. 24935167

[ajpa70137-bib-0003] Brůžek, J. , F. Santos , B. Dutailly , P. Murail , and E. Cunha . 2017. “Validation and Reliability of the Sex Estimation of the Human Os Coxae Using Freely Available DSP2 Software for Bioarchaeology and Forensic Anthropology.” American Journal of Physical Anthropology 164, no. 2: 440–449. 10.1002/ajpa.23282. 28714560

[ajpa70137-bib-0004] Buikstra, J. E. , and D. H. Ubelaker . 1994. Standards for Data Collection From Human Skeletal Remains: Proceedings of a Seminar at the Field Museum of Natural History, Organized by Jonathan Haas. Arkansas Archeological Survey.

[ajpa70137-bib-0005] Cheverud, J. M. 1988. “A Comparison of Genetic and Phenotypic Correlations.” Evolution 42: 958–968. 10.1111/j.1558-5646.1988.tb02514.x. 28581166

[ajpa70137-bib-0006] Grabowski, M. W. , J. D. Polk , and C. C. Roseman . 2011. “Divergent Patterns of Integration and Reduced Constraint in the Human Hip and the Origins of Bipedalism.” Evolution 65, no. 5: 1336–1356. 10.1111/j.1558-5646.2011.01226.x. 21521191

[ajpa70137-bib-0007] Haber, A. 2011. “A Comparative Analysis of Integration Indices.” Evolutionary Biology 38, no. 4: 476–488. 10.1007/s11692-011-9137-4.

[ajpa70137-bib-0008] Hallgrímsson, B. , H. Jamniczky , N. M. Young , et al. 2009. “Deciphering the Palimpsest: Studying the Relationship Between Morphological Integration and Phenotypic Covariation.” Evolutionary Biology 36, no. 4: 355–376. 10.1007/s11692-009-9076-5. 23293400 PMC3537827

[ajpa70137-bib-0009] Jantz, R. L. , and S. D. Ousley . 2005. FORDISC 3.0: Personal Computer Forensic Discriminant Functions. University of Tennessee, Knoxville.

[ajpa70137-bib-0010] Jerković, I. , Ž. Bašić , Š. Anđelinović , and I. Kružić . 2020. “Adjusting Posterior Probabilities to Meet Predefined Accuracy Criteria: A Proposal for a Novel Approach to Osteometric Sex Estimation.” Forensic Science International 311: 110,273. 10.1016/j.forsciint.2020.110273. 32272305

[ajpa70137-bib-0011] Klales, A. R. 2018. MorphoPASSE: The Morphological Pelvis and Skull Sex Estimation Database. Washburn University.

[ajpa70137-bib-0012] Klales, A. R. 2020. “Practitioner Preferences for Sex Estimation From Human Skeletal Remains.” In Sex Estimation of the Human Skeleton, edited by A. R. Klales , 11–23. Academic Press.

[ajpa70137-bib-0013] Klales, A. R. , and K. M. Lesciotto . 2025. “Reevaluating Skeletal Sex Estimation Practices in Forensic Anthropology.” Journal of Forensic Sciences 70, no. 3: 825–834. 10.1111/1556-4029.70014. 40104904

[ajpa70137-bib-0014] Klales, A. R. , S. D. Ousley , and J. M. Vollner . 2012. “A Revised Method of Sexing the Human Innominate Using Phenice's Nonmetric Traits and Statistical Methods.” American Journal of Physical Anthropology 149, no. 1: 104–114. 10.1002/ajpa.22102. 22714398

[ajpa70137-bib-0015] Kruuk, L. E. B. 2004. “Estimating Genetic Parameters in Natural Populations Using the ‘Animal Model’.” Philosophical Transactions of the Royal Society of London. Series B: Biological Sciences 359, no. 1446: 873–890. 10.1098/rstb.2003.1437. 15306404 PMC1693385

[ajpa70137-bib-0016] Kruuk, L. E. B. , and J. D. Hadfield . 2007. “How to Separate Genetic and Environmental Causes of Similarity Between Relatives.” Journal of Evolutionary Biology 20, no. 5: 1890–1903. 10.1111/j.1420-9101.2007.01377.x. 17714306

[ajpa70137-bib-0017] Kuchař, M. , A. Pilmann Kotěrová , A. Morávek , et al. 2024. “Automatic Variable Extraction From 3D Coxal Bone Models for Sex Estimation Using the DSP2 Method.” International Journal of Legal Medicine 138, no. 6: 2647–2658. 10.1007/s00414-024-03301-4. 39102091 PMC11490455

[ajpa70137-bib-0018] Langley, N. R. , L. M. Jantz , S. D. Ousley , and R. L. Jantz . 2016. Data Collection Procedures for Forensic Skeletal Material 2.0.

[ajpa70137-bib-0019] Lesciotto, K. M. , and A. R. Klales . 2025. “Sex Estimation Using Metrics of the Innominate: A Test of the DSP2 Method.” Journal of Forensic Sciences 70, no. 1: 249–257. 10.1111/1556-4029.15645. 39460544 PMC11693525

[ajpa70137-bib-0020] Machado, M. P. S. , S. T. Costa , A. R. Freire , et al. 2018. “Application and Validation of Diagnose Sexuelle Probabiliste V2 Tool in a Miscegenated Population.” Forensic Science International 290: 351.e351–351.e355. 10.1016/j.forsciint.2018.06.043. 30077496

[ajpa70137-bib-0021] Mallard, A. M. , K. R. R. Savell , and B. M. Auerbach . 2017. “Morphological Integration of the Human Pelvis With Respect to Age and Sex.” Anatomical Record 300, no. 4: 666–674. 10.1002/ar.23547. 28297178

[ajpa70137-bib-0022] Meloro, R. , S. D. Tallman , C. G. Streed , et al. 2025. “A Framework for Incorporating Diverse Gender Identities Into Forensic Anthropology Casework and Theory.” Current Anthropology 66, no. 4: 534–560. 10.1086/736355.

[ajpa70137-bib-0023] Murail, P. , J. Brůžek , F. Houët , and E. Cunha . 2005. “DSP: A Tool for Probabilistic Sex Diagnosis Using Worldwide Variability in Hip‐Bone Measurements.” Bulletins et Mémoires de la Société d'Anthropologie de Paris 17, no. 3–4: 167–176. 10.4000/bmsap.1157.

[ajpa70137-bib-0024] Pavlicev, M. , J. M. Cheverud , and G. P. Wagner . 2009. “Measuring Morphological Integration Using Eigenvalue Variance.” Evolutionary Biology 36, no. 1: 157–170. 10.1007/s11692-008-9042-7.

[ajpa70137-bib-0025] Quatrehomme, G. , I. Radoman , L. Nogueira , P. du Jardin , and V. Alunni . 2017. “Sex Determination Using the DSP (Probabilistic Sex Diagnosis) Method on the Coxal Bone: Efficiency of Method According to Number of Available Variables.” Forensic Science International 272: 190–193. 10.1016/j.forsciint.2016.10.020. 27856048

[ajpa70137-bib-0026] R Core Team . 2025. R: A Language and Environment for Statistical Computing. R Foundation for Statistical Computing.

[ajpa70137-bib-0027] Rolian, C. 2014. “Genes, Development, and Evolvability in Primate Evolution.” Evolutionary Anthropology 23, no. 3: 93–104. 10.1002/evan.21409. 24954217

[ajpa70137-bib-0028] Santos, F. , P. Guyomarc'h , E. Cunha , and J. Brůžek . 2020. “DSP: A Probabilistic Approach to Sex Estimation Free From Population Specificity Using Innominate Measurements.” In Sex Estimation of the Human Skeleton, edited by A. R. Klales , 243–269. Academic Press.

[ajpa70137-bib-0029] Sharma, K. 2002. “Genetic Basis of Human Female Pelvic Morphology: A Twin Study.” American Journal of Physical Anthropology 117, no. 4: 327–333. 10.1002/ajpa.10055. 11920368

[ajpa70137-bib-0030] Spradley, M. K. , and R. L. Jantz . 2011. “Sex Estimation in Forensic Anthropology: Skull Versus Postcranial Elements.” Journal of Forensic Sciences 56, no. 2: 289–296. 10.1111/j.1556-4029.2010.01635.x. 21210801

[ajpa70137-bib-0031] Thomas, R. M. , C. L. Parks , and A. H. Richard . 2016. “Accuracy Rates of Sex Estimation by Forensic Anthropologists Through Comparison With DNA Typing Results in Forensic Casework.” Journal of Forensic Sciences 61, no. 5: 1307–1310. 10.1111/1556-4029.13137. 27352918

[ajpa70137-bib-0032] Winburn, A. P. , A.‐D. Yim , and M. K. Stock . 2022. “Recentering Forensic Anthropology Within a Multifaceted Body of Evolutionary Theory: Strengthening Method by Making Theory Explicit.” American Journal of Biological Anthropology 179, no. 4: 535–551. 10.1002/ajpa.24628.

[ajpa70137-bib-0033] Xu, L. , E. Kun , D. Pandey , et al. 2025. “The Genetic Architecture of and Evolutionary Constraints on the Human Pelvic Form.” Science 388, no. 6743: eadq1521. 10.1126/science.adq1521. 40208988

